# Nurses’ workarounds in acute healthcare settings: a scoping review

**DOI:** 10.1186/1472-6963-13-175

**Published:** 2013-05-11

**Authors:** Deborah S Debono, David Greenfield, Joanne F Travaglia, Janet C Long, Deborah Black, Julie Johnson, Jeffrey Braithwaite

**Affiliations:** 1 Centre for Clinical Governance Research, Australian Institute of Health Innovation, University of New South Wales, Sydney, NSW 2052, Australia; 2 School of Public Health and Community Medicine and Centre for Clinical Governance Research, Australian Institute of Health Innovation, University of New South Wales, Sydney, NSW 2052, Australia; 3 Faculty of Health Sciences, University of Sydney, Sydney, NSW 2141, Australia

**Keywords:** Workaround, Violation, Deviation, Short cut, First order problem solving, Patient safety, Procedural failure

## Abstract

**Background:**

Workarounds circumvent or temporarily ‘fix’ perceived workflow hindrances to meet a goal or to achieve it more readily. Behaviours fitting the definition of workarounds often include violations, deviations, problem solving, improvisations, procedural failures and shortcuts. Clinicians implement workarounds in response to the complexity of delivering patient care. One imperative to understand workarounds lies in their influence on patient safety. This paper assesses the peer reviewed empirical evidence available on the use, proliferation, conceptualisation, rationalisation and perceived impact of nurses’ use of workarounds in acute care settings.

**Methods:**

A literature assessment was undertaken in 2011–2012. Snowballing technique, reference tracking, and a systematic search of twelve academic databases were conducted to identify peer reviewed published studies in acute care settings examining nurses’ workarounds. Selection criteria were applied across three phases. 58 studies were included in the final analysis and synthesis. Using an analytic frame, these studies were interrogated for: workarounds implemented in acute care settings by nurses; factors contributing to the development and proliferation of workarounds; the perceived impact of workarounds; and empirical evidence of nurses’ conceptualisation and rationalisation of workarounds.

**Results:**

The majority of studies examining nurses’ workarounds have been published since 2008, predominantly in the United States. Studies conducted across a variety of acute care settings use diverse data collection methods. Nurses’ workarounds, primarily perceived negatively, are both individually and collectively enacted. Organisational, work process, patient-related, individual, social and professional factors contribute to the proliferation of workarounds. Group norms, local and organisational culture, ‘being competent’, and collegiality influence the implementation of workarounds.

**Conclusion:**

Workarounds enable, yet potentially compromise, the execution of patient care. In some contexts such improvisations may be deemed necessary to the successful implementation of quality care, in others they are counterproductive. Workarounds have individual and cooperative characteristics. Few studies examine nurses’ individual and collective conceptualisation and rationalisation of workarounds or measure their impact. The importance of displaying competency (image management), collegiality and organisational and cultural norms play a role in nurses’ use of workarounds.

## Background

Workaround behaviours are those that circumvent or temporarily ‘fix’ an evident or perceived workflow block. Workplace workarounds are used to: solve problems [1,2]; sidestep ‘problematic’ rules [3]; bypass workflow blocks created by safety mechanisms [4]; address poor workflow design [5] and organisational and system issues [3]; save time [6]; backup software data applications [7]; compensate for inadequate technology [8,9]; patch software glitches [10]; or offer solutions to a range of problems including shortcomings in staffing, equipment and supplies [11]. Workarounds are claimed to increase when the complexity of the task is incompatible with the degree of structure imposed by the system [12,13] and when users feel ‘controlled’ by the system [14], with end user resistance contributing to their implementation [15].

Views about workarounds tend to polarise. On the one hand, negative conceptualisations report workarounds as a subset of errors, shortcuts, deviations and violations [3,16]. Terms such as improvisations [10,17] and innovations [18] offer more positive notions of workarounds. There is a paucity of clear and uniform definitions of these related constructs [3,16]. The definition of workarounds developed for this review has two components. *Workarounds are observed or described behaviours that may differ from organisationally prescribed or intended procedures*. *They circumvent or temporarily ‘fix’ an evident or perceived workflow hindrance in order to meet a goal or to achieve it more readily.*

Healthcare is a high-hazard industry in which workers have the potential to kill or maim [19:85]. More than most other industries, healthcare is complex, fragmented, decentralised and unevenly regulated [19] with clinicians required to learn on the job at the same time as they are required to display professional autonomy. Healthcare is characterised simultaneously by routine, highly organised and ultra safe practices (e.g. blood product protocols) and unpredictable, erratic hazardous demand. It is comprised of both long-term patient-clinician relationships (e.g. chronic disease) and acute, fleeting interactions (e.g. outpatient and emergency department episodes) [20]. These features of healthcare shape the way people work, behave and respond to the demands of clinical practice. Rules, policies and technologies seek to standardise clinicians’ practice. Clinicians seem to implement workarounds as a way of responding to the complexity of care within a system that increasingly demands standardisation. Although nurses are touted the masters of workarounds [21] the empirical literature focusing on them has been slow to flourish [16]. Nurses comprise the majority of the healthcare workforce. Therefore while acknowledging the corpus of literature on workarounds in other industries [22], this study focuses on nurses’ behavioural workarounds in acute healthcare organisations.

One imperative to understand workarounds in healthcare lies in their influence on safe care. Workarounds can both subvert and augment patient safety. In circumventing safety blocks [4], masking deficiencies [23,24], and undermining standardisation [25], they potentially jeopardise care to patients. Conversely, workarounds operate as localised acts of resilience [26,27], are at times crucial to the delivery of services [4], operate as adaptions to inefficiencies [20] and provide opportunities for improvement [28].

To enervate the negative and harness the positive potential of workarounds in healthcare, we must firstly understand the factors that influence their implementation and proliferation and the role of local and organisational culture in shaping them. This premise underpins this review, the purpose of which is: to assess the peer reviewed empirical evidence available on the use, proliferation and perceived impact of workarounds by nurses in acute care settings; and to examine how they are conceptualised and rationalised by those who use them.

Given the significance of the topic and the increase in publications in this area since 2008, it is timely to review the literature on workarounds. A 2008 review of workarounds in healthcare settings concluded that because there are so few studies that have empirically studied work-arounds “it was not possible to produce a typical quantitative review of the literature” [16:3]. A 2009 review of the empirical literature examining a construct overlapping with workarounds, rule violations in work settings, identified that this too is an area requiring further work [3]. Since the publication of these literature reviews significantly more work has been published in this area (Table 1).

**Table 1:** Scopus search using search term (workaround* OR work-around) [accessed 5^**th **^**March 2012]**

**Year**	**Number of references identified in Scopus**
2008-2012 (<4 years)	517
2000-2007 (7 years)	429
1961-1999 (38 years)	251

## Method

### Scope

While our paper narrows the focus of Halbesleben et al’s 2008 review [16] to workaround behaviours of nurses, it also broadens the enquiry to examine literature from a wider range of disciplines including Safety Science and Sociology. It also includes a greater variety of search terms to capture empirical literature on behavioural workarounds used by nurses. We differentiate from the work by Alper and Karsh [3], by narrowing the focus to nurses’ behavioural workarounds (including situational violations). Our study builds on both reviews by also examining nurses’ collective and individual conceptualisation and rationalisation of workarounds. These criteria allow for a more detailed and nuanced examination of workarounds.

**Figure 1:**
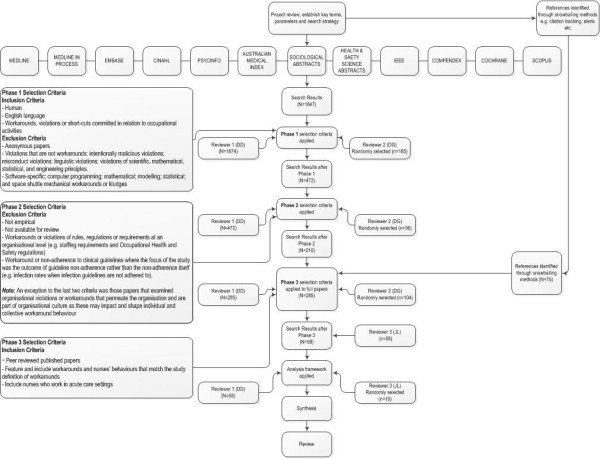
The literature review process.

Scoping methodology offers an opportunity to develop an understanding of multiple perspectives on a single issue [29]. We adopted a similar approach used in other studies [30] and diverged from the methodology described by Levac and colleagues [31] by excluding the final step of a six step framework, stakeholder consultation, which was not relevant for this study. We conducted a scoping review for several reasons. Workarounds are not yet a clearly indexed concept in academic literature databases. A systematic review involves a clearly defined topic and question. The examination of workarounds and safety violations is a pluralistic and expanding area informed by methodologically diverse research. The findings of these disparate methods do not easily lend themselves to traditional systematic reviews and meta-analyses [32]. The scoping method involves review, analysis and synthesis of a broad scope of literature. Unlike systematic reviews, scoping studies do not assess the quality of studies [31] and as they require the literature to be analytically reinterpreted they differ from narrative literature reviews as well [31]. This method is appropriate given the complexity of the area and the aim, which is to build a comprehensive picture of workarounds, rather than to weigh up the levels of evidence in relation to a specific question. The process is outlined in Figure 1.

### Search strategy

A multi-method search strategy was employed. The snowball method and reference tracking were used in conjunction with a systematic search of academic databases. The snowball method included checking references of relevant papers, serendipitously identified references, alerts and citation tracking. Studies identified in this manner up until 30th May 2012 were included for analysis.

Academic literature databases, initial search terms, limiters, inclusion and exclusion criteria were determined a priori utilising brainstorming and mind mapping techniques. An iterative process was then employed involving a preliminary review of key references and discussion with experts in literature searching techniques to hone search strategies and terms. References in articles that met the selection criteria were then searched as a way of identifying seminal articles and then tracking papers that had cited these references [3]. Key words, controlled and uncontrolled index terms in relevant references identified discipline-specific search terms were used. Consultation with a specialist university research librarian in August 2011 confirmed the search strategy and provided expertise and advice. The academic literature databases searched included: Medline; Medline in Process; Embase; Cinahl; PsycInfo; Australian Medical Index; Sociological Abstracts; Health and Safety Science; IEEE; Compendex; Cochrane Database of Systematic Review; and Scopus. All databases were interrogated using the search terms workaround*/work-around*/’work around’; and Violation* + Safety + Rule*/Policy. In addition, Medline; Medline in Process; Embase; Cinahl; PsycInfo; Australian Medical Index; Sociological Abstracts; Health and Safety Science data bases were searched using the search terms: short-cut*/shortcut*; violation*; problem-solving; ‘temporary fix*’; ‘informal practice*’; ‘informal interaction*’; ‘creative solution*’; deviation*’; and ‘procedural error*’ cross-tabulated with nurs*.

Search terms were subjected to standardised procedures. Truncation of the search term allowed for the search of plurals and other suffixes. Enclosing the search term within quotation marks restricted the search to the exact phrase. Limiters “human” and “English language”, “NOT prison OR parole” were used when available.

Following the removal of duplicate and non-English references 1847 references remained. References were examined against the selection criteria as outlined following.

### Selection criteria

Selection criteria were developed both a priori and through an iterative process that involved examination of the references and discussion between two authors (DD and DG) across three phases. At each phase, the selection criteria were refined to capture only those studies relevant to the review objective (Figure 1). Post hoc development of selection criteria is an integral part of the scoping review process [30]. In Phase 1 the selection criteria were broad to include papers examining workarounds, violations or short-cuts committed in relation to occupational activities. Additionally, the selection criteria were designed to screen out papers examining violations that are not workarounds: intentionally malicious violations (e.g. physical, sexual and human rights violations); misconduct violations (e.g. sporting, contract, copyright, privacy and parole violations); linguistic violations; violations of scientific, mathematical, statistical and engineering principles. We excluded papers examining: software-specific; computer programming; mathematical; modelling; statistical; and space shuttle mechanical workarounds or kludges. We also excluded papers at this phase that were not written in English.

The purpose of Phase 2 was to further exclude papers if they met additional screening criteria. That is, papers that examined: workarounds or violations of rules, regulations or requirements at an organisational level (e.g. staffing requirements and occupational health and safety regulations); and workarounds or non-adherence to clinical guidelines where the focus of the study was the outcome of guideline non-adherence rather than the non-adherence itself (e.g. infection rates when infection guidelines are not adhered to). An exception to these criteria was those papers that examined organisational violations or workarounds that permeate the organisation and are part of organisational culture. These were included as they may impact and shape individual and collective workaround behaviour.

Following application of selection criteria in Phases 1 and 2, there were 210 references identified through academic database searches remaining. In addition, 75 references had been identified as relevant at face value via snowballing. The Phase 3 selection criteria were applied to these 285 references. Full papers were scrutinised and included if they met the following criteria: peer reviewed published papers; featured and included workarounds and nurses’ behaviours that matched our definition of workarounds; and involved nurses who worked in acute care settings. We adopted a conservative approach, including rather than excluding studies. There remained 44 papers identified through academic database searching and 14 identified through the snowball technique that were eligible for inclusion in the review. Two authors (DD and JL) independently examined the 58 remaining papers against the selection criteria and were in agreement regarding their inclusion.

### Analysis and synthesis

An analytic frame reflecting the objective of the study was developed by two of the authors (DD and DG) (see below). The first author (DD) used the analytic frame to interrogate all of the papers that met the selection criteria (N=58). Using a random number generator, 10 of the included studies were selected. A second author (JL) interrogated these 10 studies (17%) independently using the analysis framework. The two authors (DD and JL) compared their findings. The authors were in agreement on the extracted data. The findings are organised into categories based on the analysis framework [33]: workarounds implemented in acute care settings by nurses; factors contributing to the development and proliferation of workarounds; the perceived impact of workarounds; and empirical evidence of nurses’ conceptualisation and rationalisation of workarounds.

### Analytic frame

•Citation

•Year of publication

•Year study was conducted

•Country of study

•Study setting

•Study objective

•Participants

•Methods

•Main findings and conclusions in relation to workarounds

•Technology involved (yes, no, type)

•Definition of workarounds

•Workarounds implemented

•Development and proliferation of workarounds

•Perceived impact of workarounds

•Conceptualisation and rationalisation of workarounds

## Results

### Key study features

Just over half of the studies reviewed (59%) were published between 2008–2012, with the mode being 2009 (n=9). Empirical evidence on workarounds arises predominantly from studies conducted in acute care settings in the United States of America (USA) (n=29). The United Kingdom (UK) (n=8) [34-41], Australia (n=5) [42-46], The Netherlands (n=5) [47-51], Canada (n=3) [52-54], Japan (n=1) [55], Lebanon (n=1) [56] and Thailand (n=1) [57] also hosted studies examining workarounds. Additionally, four studies were conducted in both Canada and the USA [1,24,58,59] (Table 2).

**Table 2:** Country and setting in reviewed studies

**Country of study**		**Study setting**	
United States of America	[4, 6, 60, 61, 62*, 63, 64, 65*, 67, 68, 69*, 70–73, 74*, 75, 76, 77*, 78–81, 82*, 83–87]	Intensive Care Units	[4,35,40,58,59,61-66]
Not specified	[66]	Medical and surgical Units	[1,4,34,35,40,45,46,48],[58,59,62,64,66-70]
United Kingdom	[34-41]	Oncology Units	[1,34,41,50,58,59,62,64],[65,84]
Australia	[42-46]	Maternity Units	[1,58,59,62,66,67,71]
The Netherlands	[47-51]	Cardiovascular Units	[1,34,40,45,50,53,58,59],[66]
Canada	[52-54]	Operating Theatre	[38,52,62,72,77]
Canada and United States	[1, 24*, 58, 59*]	Emergency and trauma Units	[35,57,58,62,73-75]
Japan	[55]	Psychiatry Units	[66]
Lebanon	[56*]	Long term care Units	[6,84]
Thailand	[57]	Neurology Units	[46,51,78]
		Pediatrics	[35,40,53,54,58,59,63-66,71],[77]
		Other	[40,46,51]
		Veteran Affairs Medical Centers/Hospitals	[6,76,80,81,84]
		Community Hospitals	[1,58,59,62,72,79]
		Tertiary Hospitals	[58,64,67]
		Teaching/University/Academic Hospitals	[1,34,46,48,49,52-54,56,58],[59,64,67,68,71,75,77-79,82],[84]
		Non Academic/Non Teaching Hospitals	[34,40,58,72]

* Authors contacted.

Study settings comprised hospitals that provided general medical, specialised paediatric and psychiatric services [60], and a variety of wards including, but not limited to: intensive care [1,4,35,58,59,61-66], medical and surgical [1,4,35,45,46,58,59,62],[64,66-70], oncology [1,50,58,59,62,64,65], maternity [1,58,59,62,66,67,71], cardiac units [1,45,50,58,59], operating theatre units [38,52,62,72], emergency and trauma departments [35,57,58,62,73-75], outpatient clinics [76] and paediatrics [1,35,40,53,54,58-60,63-66,71]. These wards were identified in academic [48,49,58,64,71,75,77,78] and non-academic [58,61,72], community [1,58,59,61,62,72,79], tertiary [45,58,64] and teaching hospitals [1,40,46,52-54,59,79], in urban [1,45,53,58,59,64,69] and rural [1,43,44,58,59,61] settings (Table 2). Our examination focused on nurses’ workarounds but a number of studies also incorporated other professional groups including doctors [4,34,37,39,48-50,53,54,56],[72-76,80-82], pharmacists [4,34,35,48,50,56,70,80],[81,83,84], information technology staff [4,56,80,81,84] and other hospital employees [60,72,75,78,79,81].

While some studies used a single data collection method (n=21), this review has identified that the majority of studies investigating this topic have engaged a multi method approach (n=37) (Table 3). A combination of interview and observation was the most frequently used multi method combination. An unusual method of data collection recorded nurses’ talk about what they were doing and thinking as they were administering medication [67].

**Table 3:** Data collection methods in reviewed studies

**Method**	**Studies**
***Discrete Method***	
Observations	[62,73]
Interviews	[36,52,80,81]
Focus group interviews	[69,86]
Questionnaire surveys	[37,39,43,44,55,60,63-65,79],[83,87]
Information system data analysis	[77]
***Multi-method***	
Interview and observation	[1, 6*, 34, 40, 41, 45, 53, 54, 56, 58, 59, 61, 66, 68, 72, 74, 75, 76*, 82, 84*]
Interview and document analysis including medication chart review	[48,51]
Interview, observation and document analysis (may include medication chart review)	[38,42,50]
Interview, observation, focus group, survey and time and motion studies	[78]
Analysis of information system data and observation	[70]
Analysis of information system data, observation and interview	[4]
Observation, clinical intervention data and medication chart review	[35]
Observation and medication chart review	[46]
Interview and collection of data from support desk and information system data	[85]
Questionnaire surveys, observations, interviews and Computer Provider Order Entry (CPOE) website review	[71]
Questionnaire surveys and observation and focus groups	[57]
Questionnaire surveys and interviews	[49]
Questionnaire surveys, interviews, process mapping, information system data and document analysis	[47]
Observation and journal narration	[24]
Self-recording by nurses as they gave medication and interviews	[67]

*Observational studies that noted inclusion of ‘complementary’ and ‘opportunistic’ interviews.

The term ‘workaround’ was defined in less than 30% (n=17) of the studies reviewed [4,34,48-50,54,56,60,61,63],[67,71,72,74,77,79,82]. Definitions of workarounds predominantly articulated intent to achieve an outcome through handling failures and exceptions in workflow [48,72,74] or by bypassing formal rules, protocols, standards or procedural codes [4,49,50,54,56,61,67]. Negative and positive views of workarounds were evident in the wording of several definitions. Positive aspects of workarounds include benefits for patients [67], increased efficiency for nurses [67] and a way for nurses to avoid harmful or unrealistic expectations [71]. Other definitions of workarounds convey a negative message with workarounds described as non-compliant [56], at risk, unsafe behaviours [79]. Definitions in two of the studies intimate the simultaneous negative and positive characteristic of workarounds [50,60]. Of those studies that defined workarounds, almost three quarters (71%) were published between 2009–2012.

We included seven studies [39,40,43,44,55,64,65] that offered definitions for violations because the definition incorporated elements common to the definition of a workaround or the described behaviours aligned with the definition of a workaround. For example, violations as *necessary* deviations such as *having* to break protocol (authors’ emphasis) [64] or shortcuts [43]. Violations were employed as a way of working around rules, regulations, policies, procedures and recommendations. Definitions of violations offered in two of the seven studies specified that violations were not intended to harm [40,43]. In other studies, definitions offered for first order problem solving and deviations matched our definition of workarounds [24,58,59,73]. In this paper we use the term workaround to refer to behaviours that match the definition in this study (e.g. first order problem solving).

#### Workarounds implemented by nurses

Papers were examined for examples of behaviours that matched our operational definition of workarounds. The majority of studies offered exemplars of workarounds (n=46). However, some did not detail workaround practices [1,38,43,47,55,60,62-64,73],[83]. While in most studies examples of behaviour were clearly workarounds, there were some studies in which it was more difficult to determine and for these it was necessary to consult the offered causes of the behaviour to determine whether it could be defined as a workaround. For example, we defined the practice of not checking the identification (ID) band as a workaround when a suggested barrier to accomplishing the goal of administering the medication is the time taken to check the ID band [46]. One study examined nurses working around the need to report errors by redefining errors [42].

### Workaround categories

Nurses’ workarounds in acute care settings have been studied predominantly in relation to technology including barcode medication administration (BCMA) features [4,6,34,51,63,65,67,68],[70,84,85], Computer Provider Order Entry (CPOE) [47-50,71,82], electronic health record (EHR) [53,54,76,80,81], smart pumps for intravenous infusion [77,86], equipment [69], test ordering [75] and pharmacy dispensing [56]. Workaround behaviours have also been examined as a response to: operational failures; time pressures; and perceived workflow restraints [1,24,58,59,61,62,72,74],[78]; expectations [44,58,59,72]; and rules (formal and cultural), policies, guidelines and regulations [36-40,42-44,46,52,55-57,64-67,73,83],[87]. We grouped these into three categories: technology; operational failures and work restraints; and policies, rules and regulations. Workarounds within these categories fall into two broad groups, those that are enacted individually and those that are enacted collaboratively. Many studies portrayed participant involvement that was both collaborative and individual and described workaround behaviours that fell into more than one category. To illustrate, scanning a patient barcode on a sticker rather than on the patient’s armband is an individually enacted workaround in response to technology and policy requirements [4].

The majority of described individually enacted workarounds involve responses to technology and policy particularly in relation to medication administration. Examples of collectively and individually enacted workarounds are provided in Table 4.

**Table 4:** Illustrative examples of workarounds

**Factors**	**Studies that provided examples of individually enacted workarounds**	**Illustrative examples of individually enacted workarounds**	**Studies that provided examples of collaboratively enacted workarounds**	**Illustrative example of a collaborative enacted workaround**
**Technology,***Characteristics of the technology that impose workflow blocks/delays*	[4,6,34,48,49,51,53,54],[63,68-71,76,77,80-82,84-86]	• In a study examining nurses use of BCMA, nurses were observed to “batch” and pre-pour medications which involves scanning medications and multiple ID bands for multiple patients before commencing medication administration [6]	[4,6,48-50,53,54,56,68,69],[71,76,80-82]	• A study examining use of a CPRS identified a paper-based workaround in which doctors write orders on paper and get the nurses to input them in the CPRS and the doctor signs the nurse-entered orders later [80]
		• In a study examining the use of a CPOE system, dead zones caused the computers to freeze so the nurses used paper lists of pertinent patient information, surgery lists, whiteboards, and other computers to enhance communication and ensure that timely care was given [71]		• There were several workarounds described in a study that compared a paper-based and electronic prescribing system. For example, in the CPOE there was a similarity between the Start and Stop orders which nurses worked around by using a STOP stamp on the paper chart to indicate that the medication should be stopped. Another workaround involved nurses writing new times for administration on the paper Kardex but not entering these new times in the CPOE because nurses were blocked from making changes to orders in the system [50]
		• In a study examining the side effects of BCMA introduction, nurses were observed to workaround scanning wristbands on patients by typing in the 7-digit number because it took less time than wheeling the medication cart into the patient’s room, the patient was isolated, did not have a band on, or the wristband barcode did not scan reliably [84]		
**Operational failures, exceptions and work restraints, ** *Issues that make it difficult to complete the task: resource and equipment issues; time; illegibility; too much or not enough information; knowledge; others’ actions*	[24,35,36,40,44,48,49,57],[59,61,65,66,69,76,78,80],[81,84,85]	• A study examining the universal precaution practices of nurses in an ED, offers several examples of workarounds including nurses re-sheathing needles to workaround the distance to the disposal container and to facilitate dislodging needles from syringes; not wearing gloves to workaround the perceived greater risk of needle stick injury if the gloves were the wrong size [57]	[24,42,48,49,59,61,67,69],[72,74,76,78,80,81]	• A study examining rework and workarounds in hospital medication administration processes reported that when nurses were unable to understand a medication order, they worked around this barrier by asking other nurses’, clerks’, pharmacists’ opinions or make a decision without calling the physician because they did not want to bother or feared repercussions from bothering the physician [61]
		• In examining the relationship between work constraints imposed on nurses and patient falls, nurses were identified to multi task, keeping mental track of where they are up to in their list of tasks (cognitive head data). To work around the constraints of too much cognitive head data, nurses use written and mental chunking schemas (e.g. visual reminders and chunking groups of tasks) [78]		
				• A study of the relationship between nurses’ work constraints and patient falls identified that nurses workaround the constraints imposed by a lack of formal handover between registered nurses and assistant nurses by informal querying of the previous care nurse about fall status and use of visual cues e.g. stickers [78]
**Rules/policies/guidelines/regulations,***Formal rules, policies, guidelines, regulations regarding delivery of care*	[4,34-36,40,41,44-46,48,49,57],[61,65,66,68-71,79,82,84,85],[87]	• A study assessing the impact of a CPOE system noted that when physicians had not yet entered medication orders in the system, nurses worked around the delay by beginning medication work based on the notes they took during medical rounds [49]	[4,6,42,48-50,52,56,67,68],[71,75,80,81,84]	• The clinicians work around the policy that requires completion of an authorisation form for a restricted antibiotic to be dispensed [56]
				• Collaboration is needed to work around error reporting by redefining the error. For example, a nurse may be given the medication chart from the day before to fix because she/he forgot to record it on their last shift [42]
		• A study examining baby feeding practices by midwives in 2 UK hospitals, identified that while feeding breast fed babies a bottle of artificial milk was not evidence-based practice and against policy, midwives secretly gave bottles of artificial milk at night, working around espoused policy requirements by calling it a 'special’ cup feed (a cup feed being acceptable to policy) [36]		

Legend: *BCMA* (barcode medication administration); *CPOE* (Computer Physician Order Entry); *CPRS* (Computerized Patient Record System); *ED* (Emergency Department).

#### Factors contributing to the development and proliferation of workarounds

We examined the studies for factors identified as leading to the development of workarounds. We also sought evidence for those factors that encourage or enable established workarounds to continue, for example, nurses sharing or teaching workarounds to junior staff [53,69].

Workarounds develop in response to factors that are perceived to prevent or undermine nurses’ care for their patients or are not considered in the best interest of their patients, make performance of their job difficult, or potentially threaten professional relationships. These factors can be categorised as organisational, work process, patient, individual clinician and relational/professional factors.

### Organisational factors

Staffing levels, the need to manage heavy and fluctuating workloads (working in crisis mode) [78] and productivity pressures were commonly offered organisational causes of workaround behaviours [4,24,42-44,51,52,58,59,62],[78,83,87]. In addition negative organisational climate characterised by poor leadership, a lack of involvement of nurses in decision-making, few opportunities for professional development and a lack of perceived human management resources and support contributed to the development of workaround behaviours [4,43,58,60,66]. Other factors include organisational expectations that clinicians multitask [52], a lack of role clarity [4,52], ambiguity [62], organisational processes that have not been re-engineered to fit with the implementation of technology [48,71], the low status of nurses [24] and organisational guidelines and group norms that prevent visible and formal expression of emotion about patients [75].

### Work process factors

An array of work process factors giving rise to workaround behaviours were identified in the studies reviewed. The mismatch between introduced technology or policies and current workflow was one of the most common causes of workaround behaviours [4,6,34,42,47-51,53,54,56],[61,63,68-71,76-78,80-82,84-86]. Operational failures including resource issues, equipment not stocked properly, documentation not completed, missing information and medications and environmental factors [1,4,35,44,57-59,61,69,72],[78,85] were also typical precursors to workaround behaviours. Similarly, heavy workloads, time constraints or attempts to increase efficiency led to workaround behaviours [4,6,24,45,49,51,57-59,67],[69,76,80-84]. Workaround behaviours were also attributed to the complexity and dynamic conditions of clinical work [72,74,80,81], including interruptions [61,68,83,84], emergencies [44,50,52,57,61,64,67,71] and the lack of availability of doctors to provide information [44,48,61,66]. Studies identified that in situations there were conflicting goals [84], or where nurses perceived particular requirements as less important, appropriate, useful or necessary, they were more likely to work around them [6,36,45,57,65,87]. To illustrate, in studies comparing medication administration workarounds across wards, not checking patient identification [64] and scanning a ‘surrogate’ wristband, which is not on a patient [6] were found to be more common in long-term care wards suggesting that the imperative to check patient identification was less because the nurses were familiar with the patients [6,64].

### Patient related factors

One of the most frequently identified motives for implementing workarounds was the need to ensure that patients received care in a timely manner [4,44,45,49,61,67,82,84],[85]. Other justifications included a perception that rules and policies are not always in the best interest of the patient [36,42,66], the importance of customising care to the need of patients [4,6,42,72,84], patient isolation [4,68,84] and unavailability [6,34] and concern about the impact of adhering to policy on patients’ perceptions (e.g. wearing gowns, gloves and masks [57] and repeatedly checking patient identification [41]).

### Individual clinician factors

Causes of workarounds located with the individual were presented by some studies. These included fatigue [79,83], cognitive load [48,78,80,81], unfamiliarity with the technology or its safety features, or a perception that they are not critical or efficient [4,80,81]. In some cases nurses unknowingly use workarounds when they are unaware of hospital policies [4]. Nurses are more likely to work around rules if they do not know the content or meaning of the rule or policy [45,55], they believe they are unnecessary [57], they do not approve of them [36] or if following a rule was perceived to carry more risk than not [57].

Workarounds in relation to a new electronic system were attributed to individual’s preferred sensory input or motor activity for a task: continued use of paper provided something to ‘hear’ (hearing the paper drop into the basket); something easy to manipulate (hand held notes); and something to ‘deliver’ [80,81]. Seniority [42,53], maturity [51] and intention to turnover [60] were linked with workaround behaviours. Psychological gratification and a heroic attitude about their ability and competence to creatively and persistently solve problems and care for their patients without having to depend on a colleague’s help, causes many nurses to workaround rather than employ second order problem solving [24]. Laziness offered by a participant is reported in one study as a contributor to circumventing a protocol [41]. However, evidence from the reviewed studies suggests that workaround behaviours reflect nurses’ attempts to deliver patient centred care when workflow processes make that difficult [48,49] and that they are more likely to bend the rules if distressed and when morale is low [43]. In their study examining nurses’ use of first order problem solving Tucker and Edmondson (2003) draw on observational data to specify that it is “not because nurses are uncommitted, lazy, or incompetent” [59:63]. Nurses are more likely to engage in second order problem solving (less likely to rely on workarounds) when they are motivated and feel psychologically safe to do so [58].

### Social and professional factors

Evidence offered by some studies suggests that workaround behaviours are influenced by relational factors. To illustrate, evaluation of the impact of CPOE on nurse-physician communication identified that whether or not nurses informally acted on verbal orders before they were entered in the CPOE was dependent on their professional relationship and trust in the physician [49]. Workarounds, described as ‘situated’ practices [48,56], are enabled by collaboration and a belief that the rules are negotiable [42,56,66].

Workarounds were used because of poor communication or to enhance communication and coordination of interrelated tasks between co-working professionals [48,72,78,81,83], to avoid possible or actual inter professional confrontation [44,58,59,61], or because of inter professional etiquette [52,66] or lack thereof (e.g. nurses being logged out of BCMA while they are still using it [84] or ignoring nurses’ input about a patient’s care [24]). An emphasis on individual vigilance and a professional expectation that nurses will solve problems contributed to workarounds being implemented [44,58,59]. This notion is captured in the words of a nurse interviewed in one of the reviewed studies, “working around problems is just part of my job” [59:61].

### Proliferation of workarounds

There was evidence from the reviewed studies that collaboration enables workarounds to continue and proliferate [42,49,54,56,66,74,81]. Enactment of workarounds relies on willingness of others to help. Kobayashi et al. (2005) indicated that a “workaround cannot be effective if the persons involved are not able or willing to perform. Initiators of workarounds take their tacit knowledge of others’ skills and abilities into account when deciding how to implement workarounds” [74:1563]. Workarounds are shared or passed on informally [40,53,54,59,69,71,86] particularly from senior to junior staff, they are observed and absorbed by other professionals and become part of the group behaviour [62].

Workarounds persist because of an emphasis on efficiency [59,62,72], an expectation that staff will solve problems [24,44,58,59,72], the autonomy of clinicians [56,62] and lack of role clarity [52]. The ambiguous nature of operational failures and the expectation that they are part of work routine [1] and the diverse relationships between causes and workarounds also contribute to their persistence [4]. When facing workflow blocks, rather than necessarily asking those best equipped to correct problems, nurses ask those who are socially close so as to protect their reputation of competence, thus perpetuating workarounds rather than engaging in second order problem solving [59]. Workarounds proliferate when human resource management activities reinforce them [60], in a culture and climate that supports unsafe practices [40,41,59], rather than reporting of them [87]. Conversely, an organisational culture that promotes psychological safety [58,59], executive dedication [85], supportive leadership and assistance with root cause problem solving [58,59,82,85], compliance checking [85], simplifying processes and decreasing ambiguity [62] will slow the propagation of workarounds.

#### The perceived impact of workarounds

While it was implicit that workarounds circumvent workflow blocks and ergo deliver care, we examined papers for explicit perceptions of the impact of workarounds. A small number of studies reported the impact of the workaround practices in terms of measured outcomes, including the estimated cost in nursing time spent on workarounds [59] and the impact of safety workarounds on occupational injuries [79]. In relation to patient safety, not checking patient identification was found to be significantly associated with making an intravenous medication administration error [46]. There were no studies that measured the positive impact of workarounds for patient safety although these were suggested by some studies [e.g. 36, 78]. For the most part, studies propose potential effects of workarounds rather than provide empirical evidence for their impact. Studies were examined for evidence of potential effects of workarounds. These are grouped according to their perceived negative or positive impact in relation to patients, staff and the organisation (Table 5). Several studies identified that workarounds could be both positive and negative [1,24,48,58,59,71,82] depending on the context [69] and the expertise of those using the workarounds [54]. More studies highlighted a negative [4,6,34,40,43,45,46,49],[51,61-64,68-70,83-87] rather than positive [42,53,60,66,67,81] impact of workarounds.

**Table 5:** The potential effects of workarounds in acute care settings for patients, staff and organisation

	**Patient**	**Staff**	**Organisation**
**Positive effects**	• Care is delivered according to the patient’s specific needs [42,67]. For example, ‘batching’ care so that the patient can get a good night sleep; giving medications early so that they won’t be four hours late [42]	• Decrease stress for manager and other staff [42]	• Workarounds may lead to better rules [66]
				• Provide excellent information for improvement efforts [81,82]
		• Increase efficiency and support work [76]		
	• Circumvent barriers to delivering care [56,67]			
	• Annotating printed paper patient information sheets rather than only viewing information in EHR, enables clinicians to acquaint themselves more with the patients [53]			
**Negative effects**	• Decrease patient safety by increasing the potential for error [4,6,34,40,41,43,45-49,51],[61-64,68-70,82-87]	• Make staff vulnerable to retribution [37,39,44,66,67]	• Prevent organisational learning and improvement through hiding problems and practices that are occurring in real time [1,6,24,47,56,58,59,72]	
	• Do not accurately reflect patient care delivery (e.g. charting a medication earlier than it was given) [6,48,61,84]	• Time consuming, erode staff time and energy or increase cognitive effort [48,49,58,59,72,74,82]	• Create problems elsewhere in the system and can lead to other workarounds [4,24,48,59,62,74]	
	• Decrease surveillance of patients [72]	• Increase the risk of occupational injuries [79]	• Directly or indirectly cost hospitals money [1,24,59]	
	• Staff work without necessary equipment [72]	• Informal teaching of workarounds is problematic because there is no clarity about what clinicians are being taught [53]		
	• Loss of information about patients [49,71,75,76,81]	• Enable staff to express emotion to coordinate and work more effectively [75]	• Contribute to a culture of unsafe practices [40,62]	
	• Create new pathways to error [81]		• Potentiate security breaches (e.g. nurses borrowing access codes and posting them for easy viewing) [69]	
**Both positive and negative effects**	• In some instances workarounds enhance patient care but they can also potentiate patient harm [4,24,48,69,71]	• Workarounds may ease and accelerate performance but increase workload [48]	• Allow the use of CPOE but hide opportunities for redesign and improvement [47]	
	• Workarounds fix problems so that patient care can continue but in not addressing the underlying problem similar problems may reoccur in relation to patient care [1,58,59]	• Help with the coordination of work and reduce cognitive load by providing solutions to recurring problems but lead to unstable, unavailable or unreliable work protocols [74]	• Allow the system to continue functioning but may lead to widespread instability [74]	
	• While one workaround may prevent medication errors (e.g. using a STOP stamp on the paper medication chart to indicate that a medication has been ceased because the stop and the start orders in the CPOE look very similar) other workarounds using the same system increase error risk (e.g. recording actual administration times on paper medication chart but not in the CPOE) [48-50]	• Fix problems so that patient care can continue but in not addressing the underlying problem similar problems will occur requiring staff to address them again [58,59]		
	• Informal handover of information to workaround the lack of formal communication channels reduced falls but may create gaps in passed on patient information [78]	• Workarounds may circumvent problematic EPR-mediated communication between staff but may also create confusion if the workaround is not explained [54]		
	• Deviations are linked with good patient outcomes (innovations) and bad patient outcomes (errors) [73]			

Legend: *EHR* (Electronic Health Record); *CPOE* (Computer Physician Order Entry).

#### Nurses’ conceptualisation and rationalisation of workarounds

Less than a third of the reviewed studies explicitly examine nurses’ conceptualisations or rationalisation of their own and their colleagues’ workaround behaviours (including rule subversion, first order problem solving, deviations, violations, error re-definition) [1,36-39,42,44,52,58,59,64],[66,69,71,82,87]. Mostly conclusions in relation to this issue are not explicit. Tension in the way workarounds are perceived by nurses emerged in the evaluation of studies. On the one hand, studies reported workaround behaviours as necessary to deliver care or in the best interest of the patient [1,6,36,42,44,56,59,66],[67,69,71,72,75,80,81,84],[86]. However, nurses also identified them as unsafe in particular contexts [69,87] and as workarounds are not legally sanctioned, some nurses perceived them as professionally risky [36,44,52,66].

Workarounds were justified through autonomy of practice [62] and rationalised in some studies as acceptable when deemed not to jeopardise patient safety [40,69,87], in emergency situations [4,42,44,67,71], when the nurse is familiar with the patient [6,41,45], when the doctors’ response is predictable [66] and when the behaviours fall within the scope of the nurse’s knowledge and skill [44,66]. However, nurses also reported that not adhering to policy undermined professional ideals and quality of care [38,87] and some workarounds were considered malpractice by nursing leaders [82].

A contradiction in the perceived relationship between workaround behaviours and competency was also evident in a few studies. Fixing problems and working around rules for the sake of the patient were linked with perceived proficiency and satisfaction [59,66] and “the ability to circumvent problems validated nurses’ confidence in their competence and professionalism” [24:129]. Rules were perceived as flexible and while on the one hand part of being a ‘good nurse’ was the ability to use one’s judgement to workaround the rules for the benefit of the patient, to do so risked colleagues’ perceptions that one was not a ‘good nurse’ [66]. As workaround behaviours are not legally sanctioned, they can be viewed poorly by colleagues [36,38] and not accommodated for by ‘mediocre’ [66] and casual or non permanent nurses [42]. Expertise and patient criticality influenced the number and type of deviations from standard protocols in a critical care environment [73].

One study provides evidence that nurses perceive workarounds and breaking protocol, both terms for violations, as different concepts. This study, investigating violations in medication administration, found that working around and breaking protocol “did not fit together as a measure, and the lack of overlap between the predictors of working around protocol and breaking protocol offer evidence that the two terms measure different concepts” [65:748]. That violations and improvisations are understood to mean different things is highlighted by the findings of two studies examining attitudes to patient care behaviours that comply, violate or improvise in relation to protocols. These report that while healthcare workers and the public view violations as inappropriate, the opposite is true for compliance regardless of patient outcome. Attitudes to improvisations were influenced by outcome for the patient [37,39]. Thus nurses perceived that improvisations were acceptable if the outcome for the patient was good. Violations on the other hand were viewed as inappropriate regardless of outcome [37,39].

## Discussion

Our findings build on and extend the work of Halbesleben et al (2008) [16] and Alper and Karsh (2009) [3]. Although the literature examining nurses’ use of workarounds has increased since 2008, there are still relatively few peer reviewed studies examining nurses’ workaround behaviours as a primary focus and most that do are located in the USA. There is considerable heterogeneity in the aim, methods, settings and focus of the reviewed studies. Some studies observe the frequency and causes of workarounds; others examine attitudes of professionals to circumvention of rules. There are few studies that examine the effect of workaround behaviours in terms of measured outcomes [16]. Workaround behaviours, for example, have been shown to consume organisational resources [59], impact on health professionals occupational health and safety [79] and patient medication safety [46]. However, for the most part, the consequences of workarounds are offered tentatively rather than being solely empirically based [16]. Workarounds have a cascading effect often impacting other microsystems [48,74] thus their effect may not be immediately evident making it difficult to harness and quantify their impact.

Contributing to the relatively underdeveloped body of healthcare research focused on workarounds, given their influence on patient safety, is the difficulty in investigating them. This underlies the use of multiple rather than single research approaches to uncover workarounds’ interwoven processes and characteristics [4]. While survey questionnaires have been employed [37,39,43,44,55,60,63-65,79],[83,87], the primary methods used in the reviewed studies included a combination of observation and interviews [1,6,34,40,41,45,53,54],[56,58,59,61,66,68,72,74-76],[82,84], which are resource intensive. In addition, the possibility for such research to identify glitches or deficiencies in technology and workers ‘breaking’ rules is fraught with potential implications, that is, financial, legal and political [88].

Workarounds both straddle and widen the gaps in health care delivery [89]. Overall they are reported negatively. There are claims that their implementation: destabilises patient safety [4,49,61,63,77]; undermines standardisation [56,62]; increases physical and cognitive workload [49,59,72,82]; hides actual practice and opportunities for improvement thus preventing organisational learning [1,6,24,58,59,84,86]; and creates further problems and workarounds [24,48,56,59,72,74]. However, other accounts of workarounds describe them as mindful behaviours [60] that provide opportunities for improvement [48] and both compromise and promote patient safety [48,53]. Nurses justify workarounds as necessary circumventions to deliver timely and customised patient-centred care in complex and highly variable systems [36,42,44,47,48,56,58,61],[66,67,69,76,80,81,84]. The potential pathways of workarounds to innovation and excellence and the connection of workarounds with resilience are being recognised [26-28,90].

Studies demonstrate that workarounds are individually or collectively enacted. When enacted as a collective process, they rely heavily on: a shared view that rules are flexible [42,56,66]; a tacit agreement to enact [42,44,52,56,66]; and an understanding of who will and will not workaround [74]. There is some evidence, from a small number of studies, that group norms [40,42,58,59,86], local and organisational leadership [58,59,82,85], professional structures [24,59,74] and relationships [49] and others’ expectations [44,56,58,59,66,74] influence the implementation of workarounds. Despite the collegial nature of nursing work and the demonstrated effect of organisational and local culture on clinicians’ behaviour and attitudes [91,92], the influence of social networks, relationships, expectations and local and organisational culture on the enactment and proliferation of workarounds is under investigated.

There are suggestions that nurses’ notions of what constitutes a ‘good’ nurse, their ideologies, knowledge and experience, influence their implementation of workarounds [24,59,66]. For example, nurses viewed problem solving as part of nursing and perceived that an ability to do so alone demonstrated competency. They reported a sense of gratification at being able to solve problems individually, protect patients and deliver care [24,59]. There is evidence that nurses justify working around rules and policies for the benefit of the patient [36,42,66]. However, the importance of adhering to protocols was considered by other nurses to be central to a professional approach to patient care [38]. Introducing technology incites ambiguity in practice and changes the meaning of nursing work [93] which may undermine confidence and threaten a professional’s image.

Workarounds continue to be ill defined [16] with less than half of the studies reviewed offering a definition for workarounds or related concepts. Those that did were primarily published since Halbesleben and colleagues’ articulation of this shortcoming in 2008 [16]. The lack of clarity may reflect the uncertainty about how workarounds are conceptualised in clinical settings and by researchers. For example, some authors suggest that workarounds lead to potential errors [34], while others propose that these behaviours are the error [52,83]. Importantly, there is lack of clarity in how nurses themselves differentiate workarounds from related constructs [65]. Contributing to the confusion is that some workarounds are viewed as normal practice, with clinicians being unaware that they are in fact workarounds. Furthermore, at times informal workarounds become sanctioned practices [48]. Imprecision in how workarounds are defined and reported poses challenges for researchers and those who would synthesise the evidence.

This scoping review identifies gaps in the literature, which offer opportunities for future research. Further studies are needed that investigate nurses’: workarounds as a primary focus; individual and collective conceptualisation of their own and their colleagues workarounds *in situ*; workaround behaviours and measured patient outcomes; team and organisational cultures on the enactment and proliferation of workarounds.

### Limitations

This review examined empirical peer reviewed studies written in English. A limitation of literature reviews is that imposed by research and publication timelines, which create a lag between those studies included in the review and new published information. While every attempt was made to capture all published papers in this area using systematic and comprehensive search strategies, some may have been missed.

The main challenge in studies of this type is that workaround behaviours are difficult to delineate from other behaviours [16]. We applied an operational definition of workarounds to behaviours described in the reviewed studies and were inclusive rather than exclusive. It is possible that we missed some workaround behaviours. Alternatively it is possible that we included some behaviours that may not be workaround behaviours. We attempted to ameliorate this effect by employing two reviewers to independently cross-examine randomly selected studies in phases one and two and all of the studies in phase three.

## Conclusion

Workarounds operate as a dichotomous trope. They enable yet potentially compromise patient care and safety. They provide and hide information about clinicians’ work. They are individually and collectively enacted. Organisational, work process, patient-related, individual, social, and professional factors, group norms, local and organisational culture, image management and collegiality influence the development, implementation and maintenance of workarounds. As nurses comprise the majority of the healthcare workforce, it is important to understand the use of workarounds in this population. Understanding nurses’ practice and their perception of workaround behaviours is at the heart of apprehending how to improve healthcare at the bedside, where care is delivered.

## Competing interests

The authors declare that they have no competing interests.

## Authors’ contributions

DD participated in the design of the study, carried out the literature search and selection process, analysed and synthesised the literature and drafted the paper. DG also participated in the design of the study, the literature selection process and helped to analyse, synthesise and draft the paper. JL helped to analyse, synthesise and draft the paper. JB, DB, JT and JJ helped to design the study and to draft the paper. All the authors read and approved the final manuscript.

## Pre-publication history

The pre-publication history for this paper can be accessed here:

http://www.biomedcentral.com/1472-6963/13/175/prepub
